# Ask about smoking, not quitting: a chronic disease approach to assessing and treating tobacco use

**DOI:** 10.1186/s13722-019-0159-z

**Published:** 2019-09-02

**Authors:** Steven L. Bernstein, Benjamin A. Toll

**Affiliations:** 1 Department of Emergency Medicine, Yale School of Medicine, 464 Congress Ave., Suite 260, New Haven, CT 06519 USA; 2 Department of Health Policy and Management, Yale School of Public Health, Yale Cancer Center, New Haven, CT USA; 3 Department of Public Health Sciences, Medical University of South Carolina, Charleston, SC USA

**Keywords:** Smoking cessation, Tobacco dependence, Chronic disease

## Abstract

Tobacco use is a chronic relapsing disease, and remains the leading cause of preventable death in much of the world. Increasingly, tobacco use, chiefly cigarette smoking, is being framed as a chronic disease, with periods of use and periods of abstinence. An implicit component of this conceptualization is that treatment—both counseling and pharmacotherapy—may be needed at various intervals for extended periods of time, perhaps over an individual’s lifetime. This would mirror the treatment of other chronic conditions, such as diabetes, hypertension, or hyperlipidemia. Yet, clinical trials of tobacco dependence treatment still generally model outcome measures in terms of cessation, abstinence, or quitting, measured at discrete time points. This reinforces the notion that smoking, or tobacco dependence, is a dichotomous condition, and that one is either “cured,” or not. Although the goal of treating tobacco dependence is to ensure long-term abstinence (i.e. “quitting”), this model is discordant with clinical reality, in which of periods of tobacco use are interspersed with periods of abstinence. Hence, the goal of treatment is to lengthen the duration of the latter, while shortening the duration of the former. In the clinical arena, this dichotomous model of tobacco use is reflected in electronic health records, where smoking is generally categorized as current, former, or never. We propose that clinicians move away from the dichotomous categorization of tobacco use, and adopt methods used to categorize the status of other chronic conditions. Specifically, biomarkers such as carbon monoxide, cotinine, and anabasine, measured at regular intervals, can provide clinicians with much clearer, clinically relevant and actionable assessments of current tobacco use by their patients. This can be done without making reference to dichotomous states such as current or former use of tobacco. In psychological terms, one can frame tobacco use in terms of states, attributes in specific situations at discrete moments in time, rather than the more durable traits.

## Background

Tobacco dependence is a chronic relapsing disease [1]. It remains the leading cause of premature death and illness in high income countries, and is increasingly a leading cause of death in low- and middle-income countries.

Because of the ubiquity of tobacco use, and its associated burden of morbidity and mortality, numerous practice guidelines, professional associations and regulatory bodies recommend routine assessment of tobacco use, often at every medical visit, with identified smokers then referred for treatment by quitlines, specialty clinics or groups, and offered prescriptions of approved tobacco treatment medications.

Interestingly, however, tobacco use is modeled by clinicians and investigators differently from that of other chronic diseases. Most chronic conditions, such as hypertension or diabetes, are considered to be in “good control” or not. In other words, the underlying condition is considered present, in varying degrees of severity or activity.

Tobacco use, in contrast, is modeled as present or absent: the individual is smoking, or has “quit.” There is some justification for this approach, insofar as there is no safe level of smoking. However, this dichotomous approach creates paradoxes in how tobacco use status is recorded in electronic health records (EHRs), and creates problems for clinicians, investigators, and patients in conceptualizing treatment and relapse. An additional concern is deception—often tobacco users report abstinence, even when smoking continues [2, 3].

The aim of this commentary is to try to reconcile this paradox, and offer an alternative approach to modeling changes in tobacco use status. This work focuses on smokers of cigarettes and other combustible tobacco products, which remain the leading forms of tobacco consumption. Similar approaches might be developed to assess exposure to secondhand smoke [4]. Further, we have not attempted to address the biochemical assessment of use of the many new alternative nicotine delivery systems (ANDS, e.g., e-cigarettes, heat-not-burn, etc.) as this is a rapidly developing field.

## Main text

A peculiar feature of the medical assessment of tobacco use is that it differs markedly from the assessment of other chronic diseases. Specifically: unlike tobacco use, the assessment of control of most chronic diseases centers around the routine measurement of a validated biomarker or biomarkers. The biomarker may be a physiologic parameter, or an assay of a relevant biomolecule. In general, values of the biomarker under a critical threshold represent adequate control of the condition; values above the threshold represent inadequate control, and may call for a change in therapy. Relevant examples are presented in Table 1.

**Table 1:** Assessment of control of common chronic diseases

Disease	Assay	Type of assessment
Hypertension	Blood pressure	Physiologic
Diabetes	Glycated hemoglobin: A1c, and daily glucose	Serum
Hyperlipidemia	Lipid profile, HDL/LDL ratio, triglyceride level	Serum
Overweight/obesity	Weight, body mass index (BMI)	Physiologic
Asthma	Peak expiratory flow rate	Physiologic
HIV	Viral load, T-cell counts	Serum

These parameters and tests are commonly recorded in electronic health records (EHRs) as stand-alone fields, which can be trended, displayed visually, and shown to patients as part of feedback and counseling strategies. They are also used to monitor the quality of care delivered by providers, practices, and systems, as part of managing the health of populations. They may also affect reimbursement strategies by payors.

The implicit assumption in this type of assessment is that the condition is *always* present, but in varying degrees of control. The goal is to optimize the treatment of the condition so that these pertinent surrogate physiologic variables remain optimized, indicating the risk of sustaining an adverse clinical event (like a heart attack, stroke, or renal failure) is minimized.

Tobacco use, in contrast, is typically assessed with self-reported questions that dichotomize the condition as present, or absent. The assessments are generally variations of questions such as “Have you had a cigarette, even a puff, in the last 7 days?” If the answer is no, the patient is considered a never smoker, or former smoker; if yes, then the patient is considered still smoking, or at least having had a “slip” or “relapse.”

This dichotomous approach to assessing ongoing tobacco use is discordant with the nature of a chronic relapsing disease. A patient is not diabetic in October if the glycated hemoglobin is within the normal range, and diabetic once again in December if the HbA1c is elevated. Rather, a patient has diabetes, the control of which will vary over time.

The error in clinical reasoning lies in assessing tobacco use with a transition scale, rather than a scale that assesses current use only. In other words, labeling a patient an ex-smoker necessitates referring to an earlier state. The transition scale concept, introduced by Feinstein and Wells [5, 6], measures a change in a condition or symptom of interest, by referencing a past state. For example, pain may be described as “much better,” “a little better,” “no change,” “a little worse,” or “a lot worse.” Alternatively, it may described with a scale that references only current pain, such as a 100 mm Visual Analog Scale or an 11-point (0–10) Numerical Rating Scale. Either a transition scale or a single-state scale may be reasonable.

But labeling someone an ex-smoker, whether by self-report or a negative response to recent use of tobacco products, imputes a durability to the state of “ex-smokerness” that may not last. And, if a relapse occurs, both clinician and patient may wonder if the patient ever truly was an “ex-smoker.” Both parties may find this frustrating.

Another clinical analogy may be found in oncology. Patients who complete treatment are not considered “cured” of cancer, or “cancer free,” for at least several years. They may be in remission, certainly, but cure implies a durable state, the achievement of which may not be known for some time. So it is with tobacco use. Smokers who are tobacco-abstinent for several weeks, months, or even a year or two may be considered to be “in remission” from tobacco use. But whether they are “cured” of smoking, i.e. have become ex-smokers, will not be known for many years. Even individuals who are tobacco-abstinent for 5 years may have a relapse rate of 25% [7].

A more straightforward approach to the assessment of tobacco use that reflects the assessment of other chronic diseases could also use biomarkers. Fortunately, there are several from which to choose. These include exhaled carbon monoxide, cotinine, a nicotine metabolite (which may be measured in blood, urine, saliva, or hair), and anabasine, which is a tobacco-specific alkaloid.

Each assay has distinct advantages and disadvantages. Carbon monoxide (CO) is a product of tobacco combustion, and is commonly found in the exhaled breath of smokers. It can be measured with portable, handheld detectors. There are several limitations of CO measurement: one needs to purchase specialized detectors (which require calibration and disposable pieces that need to be purchased in an ongoing fashion); there is a relatively short half-life of breath CO (249–320 min while breathing room air [8]); baseline lung function may affect CO levels [9]; its absence in the breath of users of smokeless tobacco; and its presence from other types of exposure (marijuana smoke, car exhaust, and fire). An additional drawback is that CO measurements, absent a wireless electronic interface, clinical or ancillary staff would need to enter the readings into the EHR.

Cotinine, a metabolite of nicotine, can be measured in saliva, serum, urine, and hair. It is highly specific for nicotine use. However, it involves obtaining a sample and sending it to a clinical laboratory, with the attendant cost and manpower needed. In addition, individuals who are using pharmacologic forms of nicotine (e.g., nicotine patch) may have positive assays, even in the absence of ongoing tobacco use. In addition, there is some uncertainty as to appropriate cutoff values to demarcate smoking from non-smoking [10]. Precision medicine approaches might be needed to demarcate smoking abstinence for individual patients.

Lastly, anabasine is an alkaloid found in tobacco [11]. However, unlike cotinine, it is not present in FDA-approved nicotine replacement products. Hence, it is specific for tobacco use. The primary barrier to more widespread use of anabasine is the cost of the assay; in addition, it is not easily available in clinical laboratories.

So, how might assessment of tobacco use work? Insofar as tobacco use generally begins in adolescence, one approach would be to screen all adolescents and young adults for tobacco use of any type, with universal assay of cotinine or exhaled carbon monoxide. For individuals who screen positive, follow-up sessions with a healthcare provider or care coordinator would be indicated, to discuss whether counseling or pharmacotherapy is needed (there may be a small number of individuals with positive screens who do not smoke; for example, a parking garage attendant who is exposed daily to carbon monoxide-containing exhaust from automobiles). One appealing feature of this paradigm is that treatment would be offered earlier and more often than with current practice. Older adults who have been biochemically confirmed as nonsmokers would not, in general, require ongoing screening. In addition, parents and caregivers of infants and children could be similarly screened in pediatric settings [12].

Then, for those patients who are tobacco users, one would measure the chosen biomarker at subsequent visits, and record the result in the electronic health record (EHR). Tobacco users with low CO levels or undetectable cotinine could be praised for their efforts and encouraged to continue. Those who screen positive could be offered additional treatments, or an intensification of current treatment. Much less attention, if any, would need to be paid to the traditional dichotomous question addressing tobacco use. Also, similar to the data captured for patients with other chronic diseases, the tobacco biomarkers could be displayed graphically, shown to patients, and used by health systems to better understand the management of tobacco dependence by their own providers and practices.

One limitation of this approach that may need further research is that, in contrast to the physiologic parameters and biomarkers displayed in the table, there is no clear dose–response relationship between the magnitude of tobacco biomarkers and clinical risk. In other words, the higher one’s blood pressure, the higher the risk of stroke; the higher one’s HIV viral load, the higher one’s risk of opportunistic infection. That is not necessarily true with measurements of cotinine or exhaled carbon monoxide. For these tobacco measures, a simple dichotomous outcome would help delineate current use from current non-use. However, given the multitude of diseases related to smoking, offering treatment to all patients who smoke will benefit both the patients and the health care system immeasurably. In addition, to the extent that there is no safe level of tobacco use, dichotomizing tobacco-relevant biomarkers to indicate use or non-use is clinically sensible.

Healthcare systems may have cost and resource concerns about routine biomarker assessment of tobacco use. We would contend that the economic costs of smoking, from a societal perspective, far outweigh the cost of biochemical verification. Carbon monoxide assessment, in particular, is fairly inexpensive, consisting of the fixed costs of the monitors and calibration equipment, and marginal costs of the disposable mouthpieces. In addition, as payment plans move increasingly toward rewarding outcomes, quality, and value (i.e., value based healthcare), the routine assessment and treatment of tobacco use becomes increasingly attractive. To address workflow concerns, it might be reasonable to denote patients who self-report ongoing tobacco use as smokers, and omit biochemical testing. Individual healthcare systems can tailor the algorithm to suit their needs, patient populations, and budgets.

## Conclusions

Although it is true that one may “quit” smoking forever, and this is the optimal clinical goal, the stark reality is that most patients do relapse, with most relapses occurring within days of the quit attempt [13]. Instead of contextualizing tobacco dependence as a dichotomous condition, and the complex process of treatment as a dichotomous event, we have the opportunity to embed the results of common, validated biomarkers of tobacco dependence into the now-ubiquitous EHRs, and reframe tobacco use status as the chronic relapsing condition that it is.

In making this important change, we will reinforce, among clinicians, health systems, and patients, that tobacco use is a recurring and remitting behavior, simplify the collection of clinically pertinent assessments of use, quit the focus on quitting, and shift our attention to ongoing treatment.

## Data Availability

Not applicable.
